# Machine Learning Modeling for ABC Transporter Efflux and Inhibition: Data Curation, Model Development, and New Compound Interaction Predictions

**DOI:** 10.1021/acs.molpharmaceut.5c01065

**Published:** 2025-10-20

**Authors:** Nada J. Daood, Sean R. Carey, Elena Chung, Tong Wang, Anna Kreutz, Mounika Girireddy, Suman Chakravarti, Nicole C. Kleinstreuer, Jacqueline B. Tiley, Lauren M. Aleksunes, Hao Zhu

**Affiliations:** † Department of Chemistry and Biochemistry, 3536 Rowan University, Glassboro, New Jersey 08028, United States; ‡ Center for Biomedical Informatics and Genomics, 12255 Tulane University School of Medicine, New Orleans, Louisiana 70112, United States; § Inotiv-RTP, Morrisville, North Carolina 27560, United States; ∥ MultiCASE Inc, Mayfield Heights, Ohio 44124, United States; ⊥ National Toxicology Program Interagency Center for the Evaluation of Alternative Toxicological Methods, National Institute of Environmental Health Sciences, Research Triangle Park, North Carolina 27709, United States; # Division of Pharmacotherapy and Experimental Therapeutics, UNC Eshelman School of Pharmacy, University of North Carolina at Chapel Hill, Chapel Hill, North Carolina 27599, United States; ∇ Department of Pharmacology and Toxicology, Rutgers University, Piscataway, New Jersey 08854, United States

**Keywords:** ABC transporters, MDR1, P-gp, BCRP, machine learning, QSAR, brain exposure

## Abstract

In recent years, multiple computational studies have used machine learning models to predict substrate binding and inhibition of ATP-binding cassette (ABC) transporters. However, many of these studies relied on relatively small training sets with limited applicability. In this study, we manually curated over 24,000 bioactivity records (i.e., inhibition, binding affinity, permeability) for the ABC transporters P-gp, BCRP, MRP1, and MRP2 from more than 900 literature sources in ChEMBL, with additional data from PubChem and Metrabase. This effort yielded eight data sets, comprising around 8800 unique chemicals with one or more substrate binding or inhibition activities for these four efflux transporters. Quantitative structure–activity relationship (QSAR) models were developed for each of the eight data sets using combinations of four machine learning algorithms and three sets of chemical descriptors. The resulting models demonstrated excellent performance by 5-fold cross-validation, achieving an average correct classification rate (CCR) of 0.764 for the substrate binding models and 0.839 for the inhibition models. Models were validated with additional compounds from DrugBank that were known substrates or inhibitors. We further analyzed how model predictions for efflux transporter activity could estimate exposure of the brain to xenobiotics. Notably, compounds predicted as P-gp and BCRP substrates were twice or more likely to have low brain exposure compared to compounds with high brain exposure. This study provides a large and curated drug transporter binding and inhibition database for computational modeling. Applicable models based on this large database for predicting transporter substrate binding and inhibition can be used to evaluate more complex drug bioactivities, such as exposure of protected tissues to chemicals.

## Graphical abstract

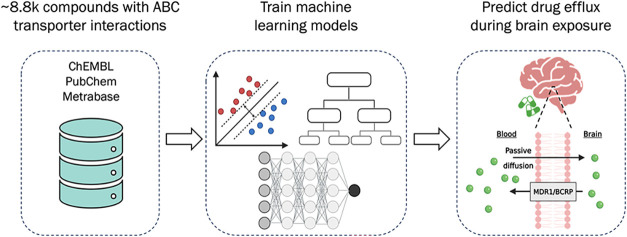

## Introduction

ATP-binding cassette (ABC) transporters are a family of membrane transporters that play a significant role in the active efflux of endogenous substances and xenobiotic compounds, like drugs and pollutants, from the cell.^1^ For pharmaceuticals, this efflux mechanism can affect their pharmacokinetic (PK) profile and propensity to cause drug–drug interactions (DDIs).^2–4^ Essential ABC drug transporters include multidrug resistance 1 P-glycoprotein (P-gp; also known as MDR1), breast cancer resistance protein (BCRP), and the multidrug resistance-associated protein (MRP) subfamily, such as MRP1 and MRP2.^5–7^ These transporters are localized to tissues that play essential roles in the absorption, distribution, metabolism, and excretion (ADME) of drugs (Table 1).^8^ They also contain ligand-binding sites that can accommodate diverse molecular structures, rendering numerous drugs susceptible to efflux. This is exemplified by the multidrug resistance to anticancer drugs conferred by ABC transporters to tumor cells. Conversely, some drugs can inhibit transporter functions, resulting in DDIs that cause the accumulation of normally effluxed chemicals, potentially leading to toxicity.^5^ For example, coadministration of P-gp inhibitors with cardiovascular drugs having a narrow therapeutic index can significantly increase the risk of toxicities.^9^ On the other hand, inhibiting P-gp can potentially improve the efficacy of anticancer drugs by increasing their concentration in target tissues.^10,11^ Thus, understanding and revealing the interplay between drugs and ABC transporters is crucial for drug development, as these interactions can significantly impact the efficacy and safety of medications.

**Table 1:** Enrichment of Major ABC Transporters across Human Tissues

	Brain^8^	Intestine^12^	Liver^13^	Placenta^8^	Kidney^14^
P-gp	√	√	√	√	√
BCRP	√	√	√	√	
MRP1				√	√
MRP2		√	√	√	√

In recent years, public databases have been established to aggregate a wider array of experimental results for diverse sets of chemicals. For example, in 2025, ChEMBL contained bioactivity records for 2.4 million compounds tested across 1.6 million assays, with manual curation of experimental results from the literature.^15^ PubChem is the largest publicly available database for chemical information, providing bioassay data and results for over 100 million chemicals.^16^ Numerous chemical-transporter interaction data (i.e., inhibition, binding affinity, permeability) can be found in these two large databases. In addition, DrugBank offers extensive information for over 16,000 drugs, including ADME profiles and transporter interactions.^17^ Moreover, transporter specific databases such as Metrabase,^18^ TP-Search,^19^ the UCSF-FDA TransPortal,^20^ the Transporter Classification Database (TCDB),^21^ and VARIDT^22^ also provide structural, functional, genomic, and/or expression information for membrane transporters. However, databases specifically recording chemical-transporter interactions, such as Metrabase and TP-Search, have not been updated in years. TransPortal, which includes chemical-transporter interactions, tissue expression data, and clinical DDIs, was recently updated in 2023 as TransPortal-TICBase.^23^ However, the number of annotated records for transporter interactions in these sources remains limited, with most data obtained from studies published before 2010. Additionally, it focuses on collecting substrate and inhibitor data, excluding potential nonsubstrates and noninhibitors, which limits its applicability in developing machine learning (ML) models. This data gap highlights the urgent need for a large and comprehensive transporter database, including newly available public transporter data that can be used for ML modeling.

ML models for drug properties/activities/toxicities, such as quantitative structure–activity relationship (QSAR) models, have emerged as valuable tools in drug discovery, offering a time- and cost-effective alternative to animal testing.^24–28^ As testing for interactions with ABC transporters is crucial, previous studies have focused on developing computational models to identify critical pharmacophores that are responsible for interactions, as summarized by a number of reviews.^29–33^ Among the four ABC transporters, P-gp is the most extensively studied, owing to its early discovery, ubiquitous expression across many tissues, broad substrate diversity, and significant influence on drug absorption and therapeutic outcomes.^34,35^ However, the computational studies for P-gp have relied on training data collected over a decade ago,^36–39^ utilized in-house experimental data that could not be shared publicly,^40,41^ or used small training sets (e.g., <50 compounds).^42,43^ Most data collection efforts for transporters were also conducted over ten years ago,^44–49^ highlighting the need for contemporary training data for ML modeling. Although the ML models developed in many of these studies had acceptable performance, their applications for evaluating new compounds and other PK properties were limited. Our group previously published a paper that integrated transporter binding potential into predictions of blood-brain barrier (BBB) permeability.^50^ However, the inclusion of transporter binding as descriptors in this study only achieved insignificant improvements in the BBB models.

In the current study, we sought to (1) curate contemporary data sets for the substrate binding and chemical inhibition of P-gp, BCRP, MRP1, and MRP2 using public data, (2) develop and analyze ML models for their ability to predict transporter interactions, and (3) integrate these models into predictions of complex drug bioactivities such as penetration into restricted tissues (e.g., brain). We used ChEMBL as the major data source for chemical interactions with human P-gp, BCRP, MRP1, and MRP2. The retrieved data were rigorously and manually curated, and experimental details from hundreds of assays were harmonized. Strict criteria and thresholds were applied to categorize chemicals as substrates, nonsubstrates, inhibitors, or noninhibitors. Additional data from PubChem and Metrabase were integrated to train QSAR models using our in-house automated QSAR pipeline.^51^ These models were validated through predicting interactions with ABC transporters for chemicals in DrugBank and employed for predicting P-gp and BCRP substrate binding for compounds with varying levels of brain exposure. This study provides a large, comprehensive, and user-friendly transporter database for building ML models. Furthermore, the applicability of transporter model predictions offers new insights into more complex biological endpoints and can assist along the drug development pipeline.

## Methods

### Data Collection and Curation

This study focused on compiling and curating bioactivity data from ChEMBL (https://www.ebi.ac.uk/chembl/, accessed April 2025) related to ABC transporter interactions. Using Python v3.11.9 and the open-source chembl-webresource-client library developed by the ChEMBL group (https://github.com/chembl/chembl_webresource_client, April 2025), over 24,000 experimental records were retrieved for human P-gp, BCRP, MRP1, and MRP2. ChEMBL annotated each record with its literature source and assay description. All records were then manually reviewed by examining each cited literature source, noting details such as cell lines, substrates, substrate concentrations, and positive controls where available. The annotation of experimental details and suggestions for substrate and inhibition thresholds followed approaches described by Montanari and Ecker,^49^ Sedykh et al.,^48^ and TransPortal.^20^ Each bioactivity record was classified as 1 (substrate/inhibitor), 0 (nonsubstrate/noninhibitor), 0.5 (inconclusive), or N.A. (not applicable), based on conservative thresholds or thresholds informed by previous curation studies.^48,49^ For example, a bioactivity record that reported a chemical with an IC_50_ of 5 μM would be labeled as 1 (inhibitor) since chemicals with an IC_50_ ≤ 10 μM were considered inhibitors. Further classification details and activity endpoints are discussed in the Supporting Information. A majority vote was then taken to determine a chemical’s final class if a chemical had multiple records. In the case of an equal number of conflicting values (e.g., one inhibitor record and one noninhibitor record), the chemical was assigned the value 0.5. Only chemicals with binary labels of 0 or 1 for substrate and/or inhibition activity were retained for model training.

### External Data Sets

External data sets were utilized to validate the model performance and evaluate the applicability of predictions. The DrugBank database provides a record of interactions between drugs and various biological targets, including enzymes, carriers, and transporters. For this study, interactions between chemicals and ABC transporters were retrieved from the target pages in DrugBank (e.g., P-gp - https://go.drugbank.com/bio_entities/BE0001032, accessed March 2025). Similar to the training sets, eight validation sets were created from DrugBank, ranging from 8 compounds (MRP1 substrates) to 197 compounds (P-gp substrates) after removing overlaps with the associated training sets (Table S1, Supplementary Excel File).

To show the utility of our substrate model predictions, the unbound brain-to-plasma concentration ratio (*K*_p,uu,brain_), which quantifies the extent of brain exposure for testing compounds, was chosen as the primary PK endpoint. The data set of *K*_p,uu,brain_ was obtained from Fridén et al., which includes experimentally measured *K*_p,uu,brain_ values from rat studies.^52^ After removing duplicates, a total of 85 compounds, primarily drugs, remained as another external prediction set for the generated models in this study.

### Chemical Curation

Chemical structures were standardized using the CASE Ultra v1.9.0.4 DataKurator tool (MultiCASE Inc., Mayfield Heights, OH). Initially, as the SMILES provided by ChEMBL and DrugBank were not canonical SMILES, we standardized them by generating the canonical SMILES using the PubChem Identifier Exchange (https://pubchem.ncbi.nlm.nih.gov/idexchange/, April 2025). Inorganic compounds were removed, and only the largest organic component was retained for mixtures. Duplicates, which were primarily comprised of stereoisomers, were removed from each of the eight training sets, with only the chemical with the highest activity being retained. In this study, only 2D chemical structures were considered to reduce the complexity of modeling and relevant computation time. Chemicals in external sets were excluded if they overlapped with the training set chemicals. If chemicals between the training set and DrugBank had conflicting values (e.g., noninhibitor in the training set and inhibitor in the external set), then the chemicals were removed from both the training set and the external set.

### Chemical Descriptors

Three sets of chemical descriptors were calculated to quantify the chemical structures and serve as variables for model training. (1) Extended-connectivity fingerprints (ECFP) are circular topological fingerprints that describe each atom’s neighborhood within 1024-bit vectors.^53^ The fingerprints were generated with a bond radius of 3 through the Morgan algorithm.^54^ (2) MACCS keys are a set of 166 binary fingerprints that represent a variety of substructures. (3) RDKit descriptors consist of 210 molecular descriptors that represent a chemical’s physicochemical properties. All the descriptors were generated with the open-source cheminformatics toolkit RDKit v2023.09.6 in Python.

### QSAR Model Development

Four ML algorithms were used to develop the QSAR models: deep neural network (DNN), random forest (RF), support vector machine (SVM), and extreme gradient boosting (XGB). These algorithms were selected because they represent a balance of classical and modern ML approaches in cheminformatics, as demonstrated in our previous work.^55–57^ Other algorithms, such as logistic regression (LR) and *k*-nearest neighbors (KNN), were used in the initial modeling process. However, preliminary modeling on the eight training sets showed that their predictive performance was considerably lower compared to the selected algorithms (DNN, RF, SVM, and XGB) (data not shown). Thus, we focused on the four algorithms that offered the strongest performance.

The DNN was built as a multilayer perceptron, a feed-forward neural network trained through backpropagation using a nonlinear activation function.^58^ The network architecture in this study consisted of an input layer with chemical descriptors representing the training set compounds, three hidden layers, and an output layer providing the predicted probabilities for transporter interactions. RF is an ensemble method that constructs multiple random decision trees, aggregating their results through a majority vote.^59^ SVM identifies the optimal hyperplane that best separates chemicals from the binary classes.^60^ XGB, a gradient boosting method, iteratively combines several weak decision trees to create a more accurate predictive model.^61^ The DNN, RF, and SVM algorithms were implemented in Python using the open-source scikit-learn v1.4.2 library, while XGB was implemented with the xgboost v2.0.3 library. Hyperparameters for all four algorithms were fine-tuned using grid search in scikit-learn, where each model was optimized by fitting different hyperparameter combinations to the training set to select the best-performing models. The various hyperparameters used in this study for optimization are outlined in detail in our previous studies.^55–57,62,63^

Eight data sets were used for training, with data for each of the four ABC transporters divided into either substrate binding or inhibition categories. For each of the eight data sets, 12 individual models were developed using different combinations of chemical descriptors and ML algorithms. The modeling process was facilitated by an in-house automatic QSAR modeling pipeline,^51^ which is available on GitHub (https://github.com/zhu-research-group/auto_qsar). A consensus model was also generated for each data set by averaging the predictions from all the individual models. Model performance was evaluated through 5-fold cross-validation, in which the data set was randomly split into five subsets. In each iteration, four subsets were combined for training, while the remaining subset served as a test set for assessing model predictivity. This process was repeated five times, ensuring that each compound in the data set was used for testing one time.

### Evaluation Metrics for Model Performance

The performance of the models was evaluated using five metrics: sensitivity, specificity, correct classification rate (CCR), positive predictive value (PPV), and the area under the curve (AUC). Sensitivity represents the proportion of correctly identified actives (true positives) out of the total number of active compounds in the data set (eq 1). Specificity, on the other hand, represents the proportion of correctly classified inactives (true negatives) out of the total number of inactive compounds (eq 2). CCR is calculated as the average of sensitivity and specificity, representing overall model performance (eq 3). PPV indicates the proportion of true positive predictions among all compounds predicted to be active by the model (eq 4). Lastly, the AUC was calculated by plotting the true positive rate (sensitivity) against the false positive rate (1 – specificity) across different classification thresholds.

1 $$\mathrm{Sensitivity}=\frac{\mathrm{True}\;\mathrm{positives}}{\mathrm{True}\;\mathrm{positives}+\mathrm{False}\;\mathrm{negatives}}$$

2 $$\mathrm{Specificity}=\frac{\mathrm{True}\;\mathrm{negatives}}{\mathrm{True}\;\mathrm{negatives}+\mathrm{False}\;\mathrm{positives}}$$

3 $$CCR=\frac{Sensitivity+Specificity}{2}$$

4 $$\mathrm{PPV}=\frac{\mathrm{True}\;\mathrm{positives}}{\mathrm{True}\;\mathrm{positives}+\mathrm{False}\;\mathrm{positives}}$$

### Applicability Domain (AD)

The applicability domain (AD) defines the scope within which the model’s predictions are considered reliable. In this study, the generated QSAR models produced predictions on a probability scale from 0 to 1, where values of 0.5 or higher indicate substrates or inhibitors, while values below 0.5 indicate nonsubstrates or noninhibitors. An AD was implemented based on the predicted probability values of a compound’s activity across the models’ predictions to improve the predictive accuracy of the models, following an approach that has been successfully applied in previous studies.^56,57,64,65^ Compounds with a predicted probability of 0.6 or higher were classified as substrates or inhibitors, while those with a probability of 0.4 or lower were classified as nonsubstrates or noninhibitors. Predictions falling between 0.4 and 0.6 were defined as “out-of-domain” and excluded from further evaluation of model performance, as these thresholds demonstrated proven success in our previous studies.^57,66^

### Variable Analysis

Key molecular substructures accounting for transporter binding/inhibition were identified using the Shapley Additive Explanations (SHAP) approach. SHAP, originating from cooperative game theory, provides a framework for interpreting model predictions by quantifying the contribution of each variable (i.e., chemical descriptor) to individual predictions.^67^ SHAP was implemented using the open-source SHAP library (v0.48.0) in Python to calculate SHAP values for the MACCS descriptors across the eight DrugBank validation sets. The MACCS descriptors were subsequently ranked according to their mean absolute SHAP values, and the substructures associated with the top-ranked descriptors were analyzed to identify molecular features that were essential for predicting transporter binding and inhibition.

### Scoring System for Brain Exposure

To evaluate exposure of the brain to chemicals, we developed a scoring system incorporating P-gp and BCRP substrate model predictions and key physicochemical properties. Compounds were assigned a score of 1 (indicating favorability for high brain exposure) for each of the following properties: a topological polar surface area (TPSA) ≤ 90 Å^2^, hydrogen bond donors (HBD) ≤ 5, and hydrogen bond acceptors (HBA) ≤ 10.^68,69^ Compounds exceeding these thresholds received a score of 0 for the respective property. Additionally, compounds were predicted for their likelihood of being substrates for P-gp or BCRP using their respective QSAR models. A predicted probability ≥ 0.5 was classified as a substrate (score = 1), while probabilities <0.5 were considered nonsubstrates (score = 0). The final brain exposure score was calculated as the sum of the TPSA, HBD, and HBA scores, minus the P-gp/BCRP substrate score, yielding a total score ranging from −1 to 3 (eq 5).

5 $$Brain\;\exp osure\;score=S_{TPSA}+S_{HBD}+S_{HBA}-S_{P‐gp\;or\;BCRP\;efflux}$$

## Results and Discussion

### Study Workflow

Figure 1 provides an overview of the study’s workflow. First, transporter substrate and inhibition data for P-gp, BCRP, MRP1, and MRP2 were initially retrieved from ChEMBL. The data underwent manual curation, followed by classifying compounds as substrates, nonsubstrates, inhibitors, or noninhibitors. The curated database was combined with compounds from PubChem and Metrabase, forming the eight data sets (Table S2, Supplementary Excel file) used to train QSAR models by using the combination of four machine learning algorithms and three sets of chemical descriptors. The resulting models were subsequently employed to predict chemical-transporter interactions for compounds from DrugBank and the brain exposure data set. Finally, compounds with predicted P-gp- and BCRP-mediated efflux potential were analyzed to assess brain exposure to drugs.

**Figure 1:**
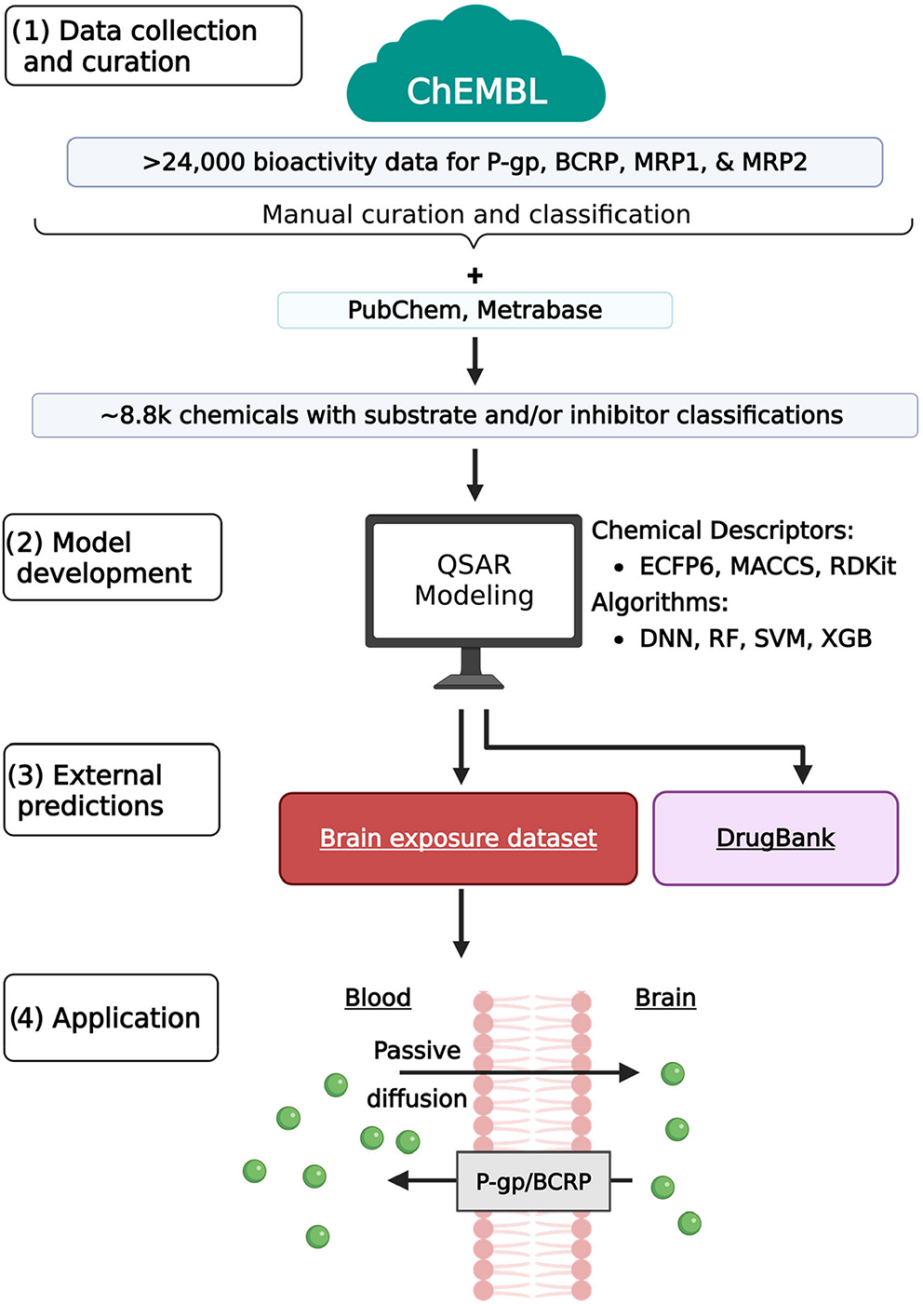
Overview of the study workflow: (1) data collection and curation, (2) QSAR modeling, (3) predictions for external chemicals, and (4) exploring brain exposure using drug efflux predictions. Created with Biorender.com.

### ChEMBL Collection and Curation

The following UniProt^70^ IDs were used to retrieve data from ChEMBL: P08183 (P-gp), Q9UNQ0 (BCRP), P33527 (MRP1), and Q92887 (MRP2). This search yielded 4394 assays from 951 sources (primarily from the literature), encompassing 24,267 *in vitro* bioactivity records related to these transporters. P-gp and BCRP are commonly tested for potential chemical interactions due to their broad substrate specificity and large ligand binding site, providing ample space for DDIs between various P-gp/BCRP substrates and inhibitors, which explains the higher number of records for P-gp and BCRP compared to MRP1 and MRP2. P-gp, as the most extensively studied transporter, accounted for the largest data set, with over 15,000 records covering more than 6300 compounds, followed by BCRP with over 5000 entries, MRP1 with 2604, and MRP2 with 1191 (Figure S1, Supporting Information). The disproportionately higher number of records for P-gp and BCRP reflects their broad substrate specificity, large and flexible ligand-binding pockets, and critical roles at key pharmacokinetic barriers (Table 1). In contrast, MRP1 and MRP2 exhibit more selective binding sites and exist in fewer organs, accounting for fewer testing data for their potential substrates and inhibitors. Furthermore, regulatory guidelines, such as those from the FDA, require preclinical assessment of P-gp and BCRP substrate and inhibition potential for DDIs, whereas no such requirements currently exist for MRP1 and MRP2.^71^

Of the more than 24,000 bioactivity records retrieved from ChEMBL, inhibition-related data comprised the majority (over 60%) and were associated with endpoints such as IC_50_, % inhibition, EC_50_, and *K*_i_ (Figure 2). IC_50_ refers to the concentration of a compound required to inhibit transporter activity by 50%, while % inhibition represents the extent of transporter inhibition relative to a control or baseline. EC_50_ is the concentration at which a compound elicits 50% of its maximal effect on transporter activity, and *K*_i_ describes the dissociation constant, indicating the binding affinity between the compound and the transporter. The “Activity” field in ChEMBL (Figure 2) often corresponds to % inhibition values reported in the original studies, and the endpoints “ratio” and “fold-change (FC)” were used variably to describe a compound’s inhibitory effect relative to a control. For substrate-related data, key endpoints included *K*_m_, the concentration of substrate where the transport rate reaches half its maximum, and apparent permeability (*P*_app_), the rate at which a substance crosses a cell membrane. Additionally, in some cases, the “ratio” endpoint in a record referred to the efflux ratio (ER), defined as the ratio of the rate at which a compound is transported out of the cell, rather than an inhibitory ratio. This variability highlights the necessity of reviewing the original literature to accurately determine whether a given record reflects inhibitory activity or substrate transport.

**Figure 2:**
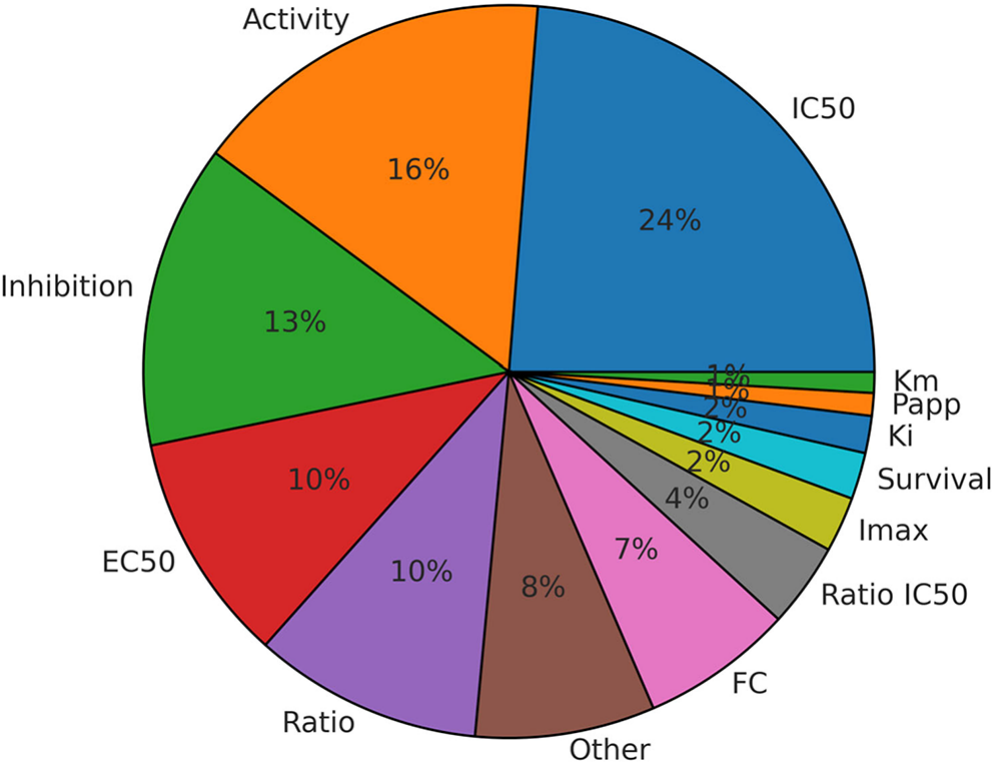
Distribution of available transporter binding data across ChEMBL for the four ABC transporters. FC—fold change; *I*_max_—maximum inhibition; *P*_app_—apparent permeability.

Experimental results for P-gp, BCRP, MRP1, and MRP2 were gathered from 733, 194, 181, and 83 studies in ChEMBL, respectively. We manually evaluated each study’s experimental protocol, interpreting results in relation to the controls used and other studies. Key assay information, such as cell lines, substrate concentrations, and positive controls, were annotated when available. In some studies, experiments were conducted to assess the potency of compounds for certain interactions with the ABC transporters without classifying whether a compound was a substrate or an inhibitor. In such cases, a classification guideline (outlined in the Supporting Information) was applied to determine the classifications for compounds. Additionally, some records in ChEMBL included comments on compounds’ classifications, which was also considered in this curation process. However, generalized comments like “active” were not considered, as they lacked specified activity type (e.g., “active” may reflect the cytotoxicity of a compound).

For the transporter substrate binding data, records were excluded if the original literature source failed to report essential experimental details, such as the type of cell line or the compound concentration. This issue was frequently encountered in studies evaluating compound efflux through P-gp and/or BCRP as part of broader *in vitro* ADME testing. For example, one study reported elcubragistat as a nonsubstrate of P-gp; however, no experimental protocol was provided to support this result.^72^ Thus, this record was excluded from our analysis of elcubragistat’s substrate binding activity. Records were also found when the ChEMBL classification as “substrate” was not consistent with the classification guideline implemented in our study. For example, progesterone (CHEMBL103) was labeled as a “substrate [+]” for P-gp in ChEMBL. However, the permeability ratio for progesterone in the associated literature source was 0.9 in the P-gp overexpressing LLC-PK1 cell line.^73^ The authors of the study also concluded that progesterone did not act as a substrate of P-gp. As a result, progesterone was classified as 0 (nonsubstrate) for P-gp.

Some specific assays, such as ATPase assays, were excluded from further evaluations as they were unsuitable for accurately classifying compounds as substrates or inhibitors. ATPase assays, often performed in insect cell membranes with human transporters overexpressed, provide insights into transporter binding affinity but can yield false positives/negatives.^74^ For example, P-gp ligands such as cyclosporine A do not consistently alter ATPase activity.^74,75^ As a result, ATPase results were excluded from final classifications. Similarly, an assay reported by Morgan et al.^76^ assessing the inhibition of human MRP2 using [3*H*]-estradiol-17beta-d-glucuronide uptake in membrane vesicles was considered the largest contributor to the MRP2 ChEMBL data set, accounting for 637 out of 1192 entries. However, known MRP2 inhibitors, such as MK571 and probenecid,^77–80^ exhibited IC_50_ values above 133 μM in this assay, which would categorize them as noninhibitors. For this reason, the data from this study were not consistent with other data sources and were thus excluded from modeling.

Additionally, our curation of bioactivity records reduced the potential misclassification of tested compounds. Specifically, several ChEMBL records reporting compounds with substrate or inhibitory activity for P-gp and BCRP were found, upon review of the primary literature sources, to originate from assays targeting other ABC transporters. For example, in the original BCRP CHEMBL data set, the compound 3-[(2-phenylpyrido­[2,3-*d*]­pyrimidin-4-yl)­amino]­benzonitrile (CHEMBL4069654) was reported to exhibit less than 25% inhibition of BCRP, which should be classified as a noninhibitor of BCRP.^81^ However, the associated assay was found to evaluate P-gp inhibition rather than BCRP inhibition, indicating that the compound was a noninhibitor of P-gp and not BCRP. As a result, this record was excluded. On the other hand, multiple records for the same compound, derived from assays targeting BCRP, confirmed the compound as a BCRP inhibitor. Thus, this compound was ultimately labeled as a BCRP inhibitor in the BCRP training set.

Discrepancies were also observed between the inhibition classifications reported in ChEMBL and PubChem. An example is the confirmatory assay for BCRP inhibition (PubChem AID: 489003; ChEMBL assay ID: CHEMBL2114819), where ChEMBL listed the compound 1-[4-[2-(3-fluorophenyl)-5-methylpyrazolo­[1,5-*a*]­pyrimidin-7-yl]­piperazin-1-yl]­ethenone (CHEMBL2130718) as “inactive” for BCRP inhibition, whereas PubChem classified the same compound as “active”. To address this inconsistency, we adopted the PubChem classification in our data set, assigning the compound as a BCRP inhibitor (labeled as 1) based on the positive bioactivity outcome reported in PubChem, rather than the “inactive” designation assigned in ChEMBL. All these above examples of data curation highlight the need for a detailed review of assay protocols and chemical classifications in public data sources, since the data (e.g., chemical classification) found in public databases can be misleading and can cause errors in the modeling process.

### Supplementing ChEMBL Data from Metrabase and PubChem for Modeling

Due to the nature of these transporter-related testing studies, there is often a bias toward reporting positive results, leading to a higher proportion of substrates or inhibitors than nonsubstrates or noninhibitors in the ChEMBL data sets (Table 2). To address this imbalance, substrate data for the four ABC transporters was collected from Metrabase, a curated transporter interaction database, to supplement the ChEMBL data for the four transporter data sets (Table 2). However, the number of BCRP nonsubstrates provided by Metrabase was insufficient to balance the training set. Thus, BCRP nonsubstrates were added from data collected by Shaikh et al. to achieve a balanced training set for generating BCRP substrate models.^82^

**Table 2:** Details of the Curated Substrate, Non-Substrate, Inhibitor, and Non-Inhibitor Data Retrieved from ChEMBL, PubChem, and Metrabase^a^

Substrate data								
	P-gp substrates	P-gp nonsubstrates	BCRP substrates	BCRP nonsubstrates	MRP1 substrates	MRP1 nonsubstrates	MRP2 substrates	MRP2 nonsubstrates
ChEMBL	303	155	66	15	55	8	41	6
Metrabase	-	166	211	160	89	90	134	121
Shaikh et al	-	-	-	116	-	-	-	-
Total	624		568		242		286	
Total (after curation)	607 (297 S/310 NS)		507 (253 S/254 NS)		182 (94 S/88 NS)		253 (134 S/119 NS)	

Inhibitor data								
	P-gp inhibitors	P-gp noninhibitors	BCRP inhibitors	BCRP noninhibitors	MRP1 inhibitors	MRP1 noninhibitors	MRP2 inhibitors	MRP2 noninhibitors
ChEMBL	2747	822	1186	333	360	718	67	583
PubChem	-	2000	-	900	-	-	-	-
Total	5569		2419		1078		650	
Total (after curation and balancing)	5282 (2493 I/2789 NI)		2395 (1176 I/1219 NI)		684 (342 I/ 342 NI)		130 (65 I/65 NI)	

a S—substrate; NS—nonsubstrate; I—inhibitor; NI— noninhibitor.

On the other hand, the MRP1 and MRP2 inhibition data sets have more noninhibitors than inhibitors, likely due to their selective binding sites and the use of their assays in studies focused on validating compound selectivity for other transporters. To balance these data sets for modeling, noninhibitors were randomly removed for the MRP1 and MRP2 inhibition data sets. For the P-gp and BCRP inhibition data sets, which had an inhibitor-to-noninhibitor ratio of roughly 3:1, noninhibitors were supplemented from high-throughput screening assays in PubChem (AIDs 1325 and 1326). From approximately 200,000 inactive compounds in each assay, 10,000 noninhibitors of P-gp and BCRP were randomly chosen and further selected if they had high structural similarity (based on Tanimoto coefficient ≥ 0.8 and MACCS keys) to inactive compounds in the training set, as described in Jiang et al.^83^ Thus, the final P-gp and BCRP inhibition data sets achieved a near 1:1 ratio (Table 2). As a result of the data curation process, we generated the largest database for chemical binding/inhibition of ABC transporters thus far. Overall, compared to the existing databases reported in previous modeling studies,^44–49,84^ the training sets in this project were substantially larger and covered a more diverse chemical space for model development and predictions.

### QSAR Model Development and Performance

The final eight curated data sets, which included P-gp, BCRP, MRP1, and MRP2 substrate and inhibition interactions (Table 2), were used to train QSAR models, producing a total of 104 models, with 12 individual models and one consensus model per data set. Using CCR as a primary indicator of model performance, 5-fold cross-validation yielded CCR values ranging from 0.685 to 0.834 for the substrate models and 0.631 to 0.935 for the inhibition models (Table 3). A total of 99 out of 104 models achieved a CCR above 0.7, reflecting excellent model performance.^85^ Additionally, the AUC scores for the individual models were satisfactory, where all the models achieved AUC scores above 0.7. Overall, the models trained on the eight data sets in this study performed well across multiple statistical metrics. Our models also demonstrated comparable or better performance than those generated from past representative modeling studies.^44,45,47–49,86^

**Table 3:** Five-Fold Cross-Validation Performance of the ABC Transporter Models^a^

			Correct Classification Rate (CCR)							
			P-gp		BCRP		MRP1		MRP2	
Algorithm	Descriptor		SUB	INH	SUB	INH	SUB	INH	SUB	INH
DNN	**ECFP6**		0.711	0.893	0.720	0.917	0.760	0.829	0.740	0.631
	**MACCS**		0.710	0.872	0.734	0.913	0.729	0.804	0.781	0.692
	**RDKit**		0.723	0.892	0.748	0.918	0.752	0.808	0.726	0.700
RF	**ECFP6**		0.685	0.839	0.714	0.898	0.820	0.827	0.783	0.731
	**MACCS**		0.732	0.849	0.749	0.919	0.785	0.806	0.796	0.646
	**RDKit**		0.734	0.877	0.757	0.916	0.823	0.864	0.814	**0.800**
SVM	**ECFP6**		0.743	0.916	0.734	0.923	0.817	0.852	0.753	0.708
	**MACCS**		0.724	0.891	0.738	0.918	0.824	0.825	0.807	0.715
	**RDKit**		0.745	0.913	0.767	0.924	0.825	0.829	0.825	0.715
XGB	**ECFP6**		0.713	0.904	0.708	0.915	0.819	0.873	0.801	0.677
	**MACCS**		0.710	0.884	0.744	0.921	0.785	0.845	0.785	0.700
	**RDKit**		0.740	0.912	0.769	0.926	**0.834**	0.855	0.786	0.785
Consensus			**0.752**	**0.924**	**0.779**	**0.935**	0.829	**0.883**	**0.833**	0.731

a CCR values in bold represent the best-performing model for the respective data set; SUB—substrate models; INH—inhibition models.

For the substrate models, all 52 models showed strong overall performance, with CCR values ranging from 0.685 to 0.834. As for the inhibition models, the BCRP inhibition models exhibited the best overall performance, where all the models achieved CCR values close to or above 0.9. The P-gp and MRP1 inhibition models also yielded satisfactory CCR values, ranging between 0.804 and 0.924. By adding an extra feature selection step into the modeling process (such as recursive feature elimination and forward selection), the modeling result showed no significant improvement (data not shown). However, four MRP2 inhibition models resulted in CCR values lower than 0.7, which is due to the low sensitivity of these models, such as the MRP2 DNN-MACCS model having a sensitivity as low as 0.385 (Table S3, Supplementary Excel File). This underperformance can be explained by the limited size of the training set (130 compounds) and reflects the need for more experimental MRP2 inhibition data for developing an enhanced model in the future.

In general, the performance of the QSAR models varied depending on the algorithm and chemical descriptors employed, as no single algorithm or descriptor consistently excelled across all individual models. However, the consensus models were the best-performing models for six of the eight data sets and demonstrated comparable performance for the remaining two data sets, making them suitable for external predictions. Importantly, the advantage of the consensus models is that they integrate predictions from multiple individual models, thereby reducing the impact of isolated false predictions. The AD was also implemented to evaluate whether model performance could be improved. Table S4 (Supplementary Excel File) showed that incorporating an AD yielded results comparable to models without an AD (Table S3). Thus, we proceeded with the assessment of external predictions without applying an AD, given the comparable performance observed.

The performance of the consensus models for external validation was evaluated using sensitivity as the primary metric, given that the DrugBank validation sets consisted of substrates and inhibitors. Among the substrate models, the BCRP consensus model demonstrated the best performance, achieving a sensitivity of 0.723 (Figure 3A). The P-gp and MRP1 substrate models exhibited moderate sensitivity values for DrugBank compounds, ranging between 0.55 to 0.65. On the other hand, among the inhibition models, the MRP1 consensus models achieved the highest sensitivity (0.956). In contrast, the P-gp and MRP2 consensus model exhibited the lowest performance (Figure 3A). Despite having the largest inhibition data set, the underperformance of the P-gp inhibition models may be due to the broad substrate specificity for P-gp, as it binds to diverse chemical structures.^87^ Furthermore, we identified inconsistencies within the DrugBank classifications: several compounds were labeled as P-gp inhibitors despite the cited literature references classifying them as noninhibitors, such as benzocaine and amodiaquine.^64,88^ The inconsistency of this data source also proved the importance of data curation for model development. In contrast, the poor performance of the MRP2 inhibition model is likely due to the limited size of the training set (i.e., only 130 compounds in the training set). Additionally, during data set curation, conflicting labels were identified for some compounds shared between the training and DrugBank validation set (e.g., indomethacin labeled as a P-gp inhibitor in DrugBank but as a noninhibitor in the training set). This suggests that certain compounds in the MRP2 inhibition set may also be mislabeled, either due to testing protocol sensitivity or differences in how transporter inhibition was classified (i.e., different thresholds being used in different sources). The chemical space and diversity of the training set also appear to influence model performance, as illustrated by the PCA plots comparing the MRP1 and MRP2 inhibition data sets in Figure 3B. Specifically, MRP1 inhibitors from DrugBank shared higher similarity to MRP1 inhibitors than MRP1 noninhibitors in the training set, which likely contributed to the high predictive sensitivity of the MRP1 inhibition consensus model. We also evaluated the effect of applying the AD in the external predictions. However, no significant improvement in predictive accuracy was observed when compounds outside the AD were excluded. This condition also indicates potential inconsistencies exist between sources in DrugBank and the training data.

**Figure 3:**
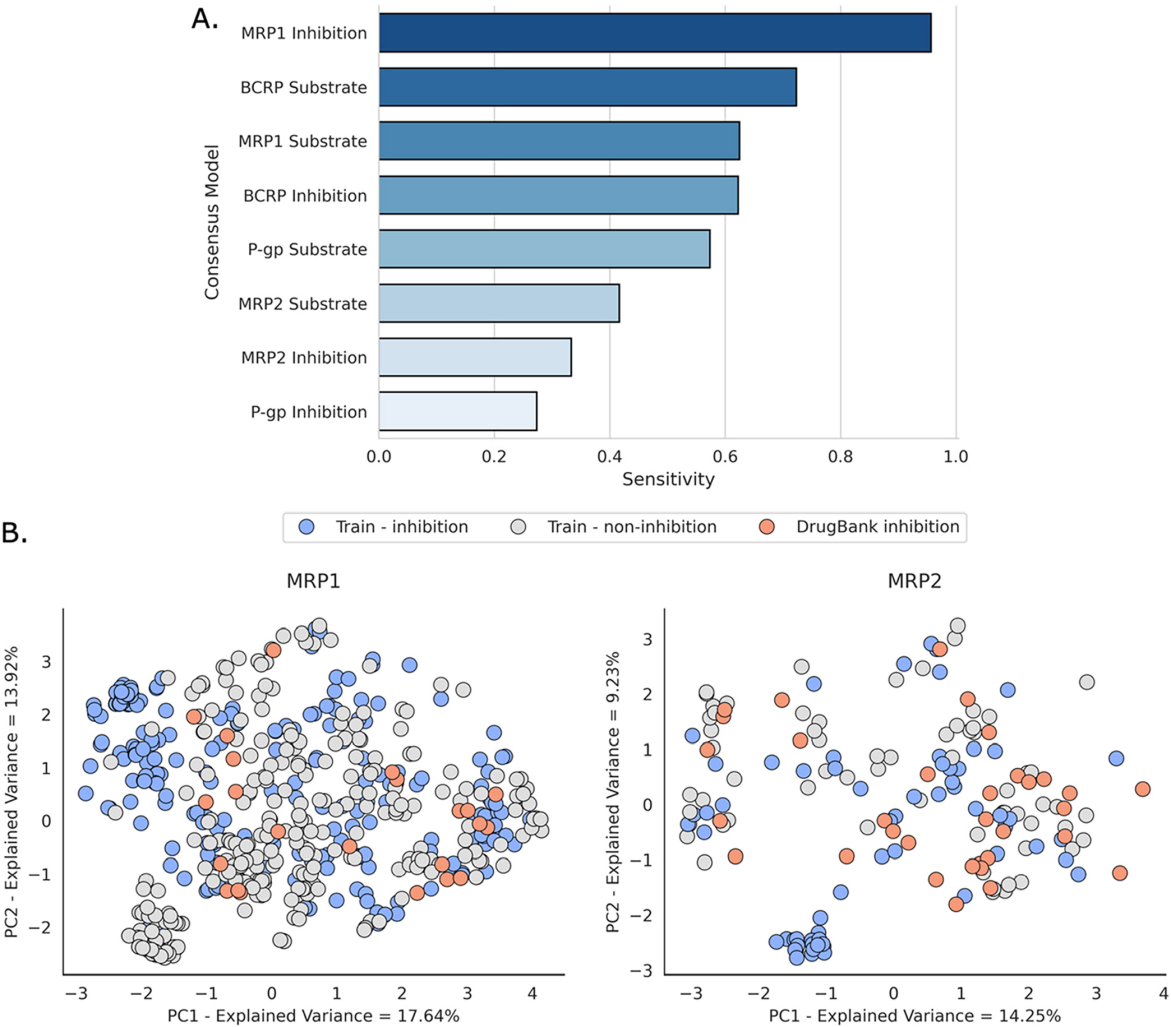
External validation of ABC transporter models using eight DrugBank data sets consisting of drugs classified as substrates/inhibitors of the four ABC transporters. (A) Model performance evaluated using sensitivity as a metric. (B) PCA plots illustrating the chemical space for the MRP1 and MRP2 inhibition training and DrugBank sets using the MACCS descriptors.

### Key Structural Features Underlying Model Predictions

Using the SHAP approach, MACCS descriptors were ranked to show their contributions to model predictions. For P-gp, substrate predictions were primarily driven by descriptors associated with polar functional groups, tertiary amines, and flexible chains and ring structures (Figure S2). On the other hand, inhibitor predictions were linked to hydrophobic substructures and multiple hydrogen bond donors and acceptors that promote strong binding within the P-gp ligand binding pocket.^89^ For BCRP, substrates were characterized by fluoride substituents, hydrogen bond donors, and planar aromatic heterocycles, while inhibitors were more strongly associated with nitrogen heterocycles, multiple aromatic rings, and extended aromatic scaffolds (Figure S3).^49^ In the case of MRP1, substrate predictions were driven by descriptors conferring increased hydrophilicity, whereas inhibitors were characterized by hydroxyl and carbonyl groups as well as tetrahedral carbons linked to three or more carbons (Figure S4).^90^ Finally, for MRP2, substrates were linked to descriptors involving multiple oxygen atoms, oxygen-containing heterocycles, and hydrogen bond donors and acceptors, while inhibitors were associated with five-membered rings, multiple heterocycles, and hydrophobic motifs (Figure S5).^91^ Overall, these descriptors highlight a number of structural features correlated with substrate recognition and inhibition, which have been partially proven by previous studies.^49,89–91^ Further mechanistic analysis and experimental validation are still needed to uncover novel molecular determinants of transporter-ligand interactions.

### The Use of Transporter Substrate Model Predictions in Brain Exposure Evaluation

The effects of drugs on the brain are determined not only by their inherent ability to cross the BBB, but also their affinity to interact with membrane transporters. P-gp and BCRP, both primary efflux transporters, are highly expressed in the brain and can significantly influence the efficacy of central nervous system (CNS) drugs. To investigate the applicability of predicted P-gp- and BCRP-mediated drug efflux to evaluate brain exposure, we applied the P-gp and BCRP substrate consensus models to predict substrate binding for compounds from a data set with measured *K*_p,uu,brain_ values (Table S5, Supplementary Excel File). The results revealed that compounds with low brain exposure (*K*_p,uu,brain_ < 0.1) were twice or more likely to be predicted as P-gp and/or BCRP substrates than compounds with high brain exposure (Figure 4A).

**Figure 4:**
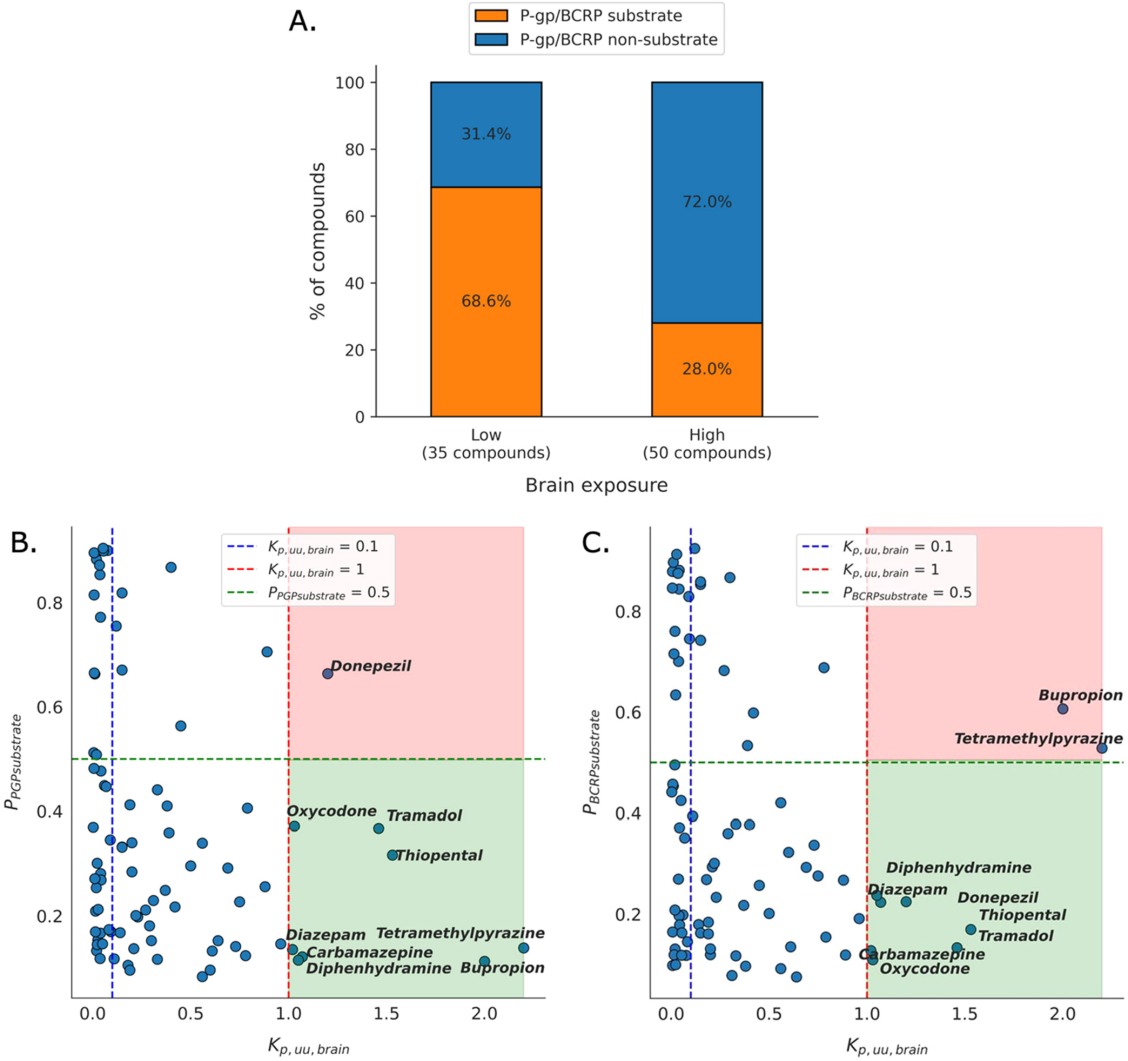
Model predictions for P-gp- and BCRP-mediated efflux of 85 compounds with experimentally measured *K*_p,uu,brain_.^52^ (A) Comparison of P-gp/BCRP substrate and nonsubstrate predictions across the 85 compounds with high (*K*_p,uu,brain_ ≥ 0.1) and low (*K*_p,uu,brain_ < 0.1) brain exposure. Scatterplots of predicted (B) P-gp and (C) BCRP substrate probabilities against *K*_p,uu,brain_ and applying different thresholds to assess brain exposure (*K*_p,uu,brain_ = 0.1 vs *K*_p,uu,brain_ = 1). Compounds in the shaded areas have a *K*_p,uu,brain_ ≥ 1, where the red area contains predicted substrates, and the green area contains predicted nonsubstrates.

The definition of high and low brain exposure varies across studies.^52,92,93^ In this study, we first assessed the relationships between the P-gp and BCRP substrate predictions and the measured *K*_p,uu,brain_ values for the 85 compounds. When applying a threshold of *K*_p,uu,brain_ above 1 to classify high brain exposure drugs, eight out of nine such compounds were predicted as nonsubstrates of P-gp (Figure 4B, green shaded area). Similarly, seven of these nine compounds were also predicted as nonsubstrates of BCRP (Figure 4C, green shaded area). The remaining three incorrectly predicted compounds (donepezil for P-gp and bupropion and tetramethylpyrazine for BCRP) were false positives, misclassified by the respective substrate models. Nonetheless, the models correctly identified the nonsubstrate status of the majority of the high exposure drugs with *K*_p,uu,brain_ ≥ 1. Notably, although the overall data set of 85 compounds comprises a mixture of central nervous system (CNS) and non-CNS drugs, all nine compounds with a *K*_p,uu,brain_ ≥ 1 were CNS drugs as classified in DrugBank, suggesting that such high *K*_p,uu,brain_ values are exclusive to drugs that penetrate the brain effectively. A review of the literature further confirmed that none of these nine drugs were reported as P-gp or BCRP substrates. Overall, these results support the prevailing hypothesis that compounds with high brain exposure are unlikely to be effluxed by P-gp or BCRP.^68,92^

When applying P-gp and BCRP substrate model predictions to evaluate drug brain exposure, it is also important to consider the physicochemical properties of target drugs that influence brain exposure. Key factors influencing *K*_p,uu,brain_ include TPSA, HBD, and HBA.^68,69,92,94^ To evaluate brain exposure using transporter binding and physicochemical properties, we incorporated P-gp/BCRP substrate predictions into a brain exposure scoring framework (see Methods, eq 5). The analysis revealed that more than 60% of the high brain exposure compounds (*K*_p,uu,brain_ ≥ 0.1) were predicted to have high exposure scores, suggesting that low binding potentials of P-gp or BCRP contributed to their favorable brain penetration (Figure 5). In contrast, among the compounds with low brain exposure (*K*_p,uu,brain_ < 0.1), 20 out of 35 compounds exhibited low exposure scores (−1, 0, or 1), of which 16 were predicted to be substrates of P-gp and/or BCRP. Furthermore, seven of the ten low exposure compounds with moderate scores (2) were also predicted as P-gp/BCRP substrates. Despite having favorable molecular properties, the active efflux of these compounds can limit their degree of brain exposure. For example, loperamide, a synthetic μ-opioid receptor agonist used to treat diarrhea, shows minimal CNS penetration due to its efflux by P-gp (*K*_p,uu,brain_ = 0.007; predicted as P-gp substrate), which limits its potential analgesic effects.^95^ Similarly, methotrexate, a chemotherapeutic agent with poor brain penetration, is actively effluxed by P-gp, BCRP, and other ABC transporters (*K*_p,uu,brain_ = 0.006; predicted as P-gp and BCRP substrate). This efflux activity contributes to its poor CNS distribution and provides a rationale for the intrathecal administration of methotrexate in the treatment of brain tumors to bypass the BBB.^96,97^ This finding provides a new strategy for applying transporter ML models to evaluate more complex drug related properties, such as brain exposure for new drug candidates.

**Figure 5:**
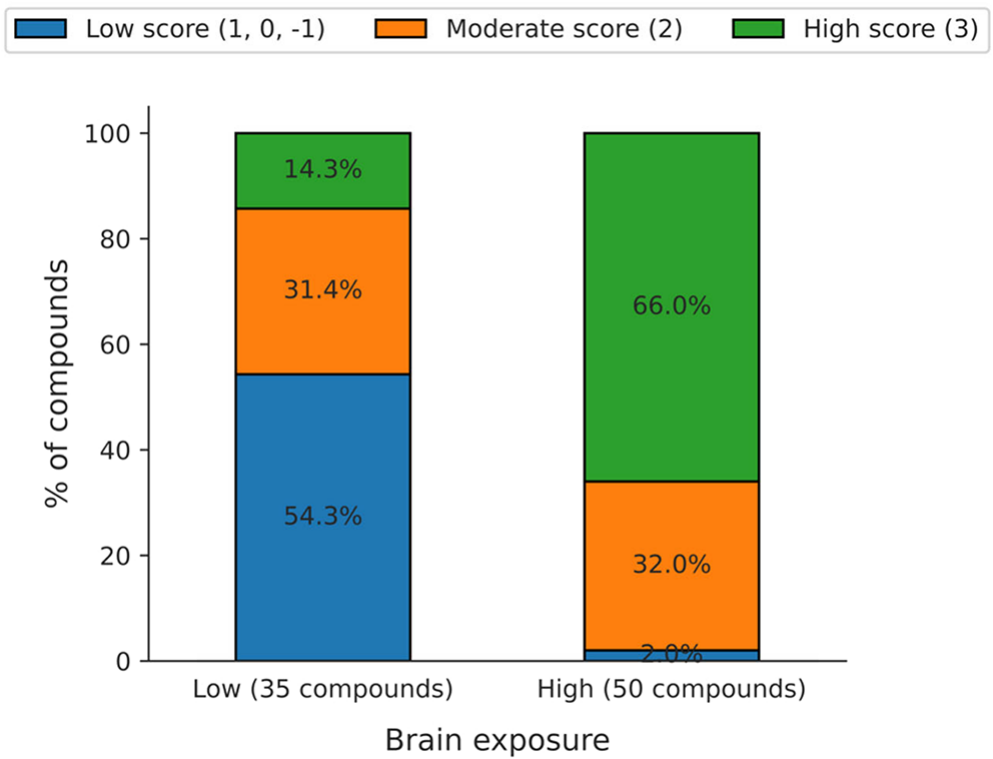
Distribution of exposure scores across the 85 compounds with high (*K*_p,uu,brain_ ≥ 0.1) and low (*K*_p,uu,brain_ < 0.1) brain exposure.

## Conclusion

This study compiled a large database of around 8800 diverse compounds with annotated and categorized transporter interaction data, including substrate binding and inhibition, for four ABC transporters. The eight training sets generated from this collection were used to build various QSAR models, most of which demonstrated good predictive performance. External validation of the developed models with new compounds highlighted the critical role of training set size and diversity, as well as the importance of thorough annotation, interpretation, and critical analysis of public data in ensuring data set accuracy. Furthermore, applying P-gp and BCRP substrate model predictions showed promising results when assessing drug brain exposure through a simple and straightforward scoring system for evaluating complex drug properties/activities *in vivo*. The inhibition models can also be applied in drug discovery to identify compounds with inhibitory activity that may interfere with the disposition of coadministered drugs, thereby enabling the assessment of DDI risks and guiding the optimization of chemical structures. Additionally, while this study focused on four key ABC transporters, the modeling framework presented in this study can be broadly extended to other transporters, like solute carrier (SLC) transporters, which are also critical in drug uptake, distribution, and elimination. Overall, this study reveals the potential of integrating ML models of *in vitro* drug properties/activities to an applicable scoring system for evaluating complex drug properties/activities *in vivo*.

## Supplementary Material





## Notes

Guidelines for classifying bioactivity records and compounds; bar plots illustrating the number of bioactivity records and unique compounds retrieved for P-gp, BCRP, MRP1, and MRP2; top 10 MACCS descriptors ranked by SHAP values for the P-gp random forest (A) substrate and (B) inhibition models; top 10 MACCS descriptors ranked by SHAP values for the BCRP random forest (A) substrate and (B) inhibition models; top 10 MACCS descriptors ranked by SHAP values for the MRP1 random forest (A) substrate and (B) inhibition models; top 10 MACCS descriptors ranked by SHAP values for the MRP2 random forest (A) substrate and (B) inhibition models (PDF)
Details and known ABC transporter interactions for DrugBank compounds; details and ABC transporter interactions for ∼8.8k compounds across eight training sets; 5-fold cross-validation evaluation metrics for the 104 generated QSAR models; 5-fold cross-validation evaluation metrics for the 104 generated QSAR models within the applicability domain; details of the brain exposure data set along with P-gp and BCRP substrate predictions (XLSX)
