# Supplementary material for: Machine Learning Modeling for ABC Transporter Efflux and Inhibition: Data Curation, Model Development, and New Compound Interaction Predictions
Source: Mol Pharm. 2025 Oct 20;22(11):7022–35. doi: 10.1021/acs.molpharmaceut.5c01065 (PMC12587445; doi:10.1021/acs.molpharmaceut.5c01065)
Supplement: Supplementary file 1 [file mp5c01065_si_001.pdf]

<u>**Supporting Information**</u>

Machine Learning Modeling for ABC Transporter Efflux and Inhibition: Data Curation, Model Development, and New Compound Interaction Predictions

Nada J. Daood[1,2], Sean R. Carey[1,2], Elena Chung[1,2], Tong Wang[1,2], Anna Kreutz[3], Mounika Girireddy[4], Suman Chakravarti[4], Nicole C. Kleinstreuer[5], Jacqueline B. Tiley,[6] Lauren M. Aleksunes[7], Hao Zhu[1,2]*

[1]Department of Chemistry and Biochemistry, Rowan University, Glassboro, NJ 08028, USA.

[2]Center for Biomedical Informatics and Genomics, Tulane University School of Medicine, New Orleans, LA 70112, USA.

[3]Inotiv-RTP, Morrisville, NC 27560, USA.

[4]MultiCASE Inc, Mayfield Heights, OH 44124, USA.

[5]National Toxicology Program Interagency Center for the Evaluation of Alternative Toxicological Methods, National Institute of Environmental Health Sciences, Research Triangle Park, NC 27709, USA.

[6] Division of Pharmacotherapy and Experimental Therapeutics, UNC Eshelman School of Pharmacy, University of North Carolina at Chapel Hill, Chapel Hill, NC 27599, USA.

[7]Department of Pharmacology and Toxicology, Rutgers University, Piscataway, NJ 08854, USA.

Corresponding Author

Hao Zhu – *Center for Biomedical Informatics and Genomics, Tulane University School of Medicine, 1430 Tulane Avenue, New Orleans, Louisiana, USA 70112;* Telephone: (504) 988-3443; orcid.org/0000-0002-3559-6129, Email: hzhu10@tulane.edu

Table of contents

Guidelines for classifying bioactivity records and compounds.

Figure S1. Bar plots illustrating the number of bioactivity records and unique compounds retrieved for P-gp, BCRP, MRP1, and MRP2.

Figure S2. Top 10 MACCS descriptors ranked by SHAP values for the P-gp random forest A) substrate and B) inhibition models.

Figure S3. Top 10 MACCS descriptors ranked by SHAP values for the BCRP random forest A) substrate and B) inhibition models.

Figure S4. Top 10 MACCS descriptors ranked by SHAP values for the MRP1 random forest A) substrate and B) inhibition models.

Figure S5. Top 10 MACCS descriptors ranked by SHAP values for the MRP2 random forest A) substrate and B) inhibition models.

Additional File: Excel spreadsheet containing Supplementary Tables 1, 2, 3, 4, and 5.

Guidelines for classifying bioactivity records and chemicals

For each transporter, the records were first sorted by the source (literature source or otherwise) from which they came from. The records were then assessed based on the assays and the experimental protocols conducted in the respective study. If the authors confirmed whether a chemical was an inhibitor, substrate, or otherwise, then the record was assigned the value 1 (inhibitor/substrate) or 0 (non-inhibitor/non-substrate). The guidelines and thresholds below were applied in the case that the authors did not specifically mention whether the result indicated inhibition or substrate binding. If the chemical's class remained unclear in the study and did not fulfill the criteria below or was outside the given threshold (e.g., $10\ \mu M < IC_{50} \leq 50\ \mu M$), then the record was assigned the value 0.5. Overall, the study's analysis of the results takes precedence over the below guideline.

A majority vote was then taken to determine a chemical's final class if a compound had multiple records. In the case of an equal number of conflicting labels (e.g., one inhibitor record and one non-inhibitor record), the chemical was assigned the value 0.5 and was excluded from the training set for modeling.

Thresholds for inhibition data:

- EC_{50}, IC_{50}, GI_{50}, $K_i \leq 10\ \mu M$ → inhibitor (1); $> 50\ \mu M$ → non-inhibitor (0)
- % inhibition ≥ 70% → inhibitor (1); < 25% → non-inhibitor (0)
- Fold-ratio inhibition ≥ 3 → inhibitor (1); < 1 → non-inhibitor (0)
- ATPase activation as an endpoint for ABC transporters is not considered as ATPase assays cannot distinguish between substrates and inhibitors, which may lead to false positives and false negatives (Bahadduri et al., 2010).
- % survival of cells ≤ 25% → inhibitor (1); > 70% → non-inhibitor (0)
- For ABC transporters, fluorescence activity ratio (FAR) ≥ 3 → inhibitor (1); < 1 → non-inhibitor.

Thresholds for substrate data:

- $K_m \leq 10\ \mu M$ → substrate (1); $> 50\ \mu M$ → non-substrate (0)
- Efflux ratios (ER) ≥ 3 → substrate (1); < 1 → non-substrate (0)
- P_{app} (apparent permeability – either basal (B) to apical (A) and apical (A) to basal (B)): Only the calculated efflux ratio will be used for classifying the compound as a substrate. The P_{app} alone does not determine substrate activity.
  - $ER = \frac{P_{app}^{B \to A}}{P_{app}^{A \to B}}$

Guidelines applicable to both inhibition and substrate data:

- K_d is a measure of binding affinity, and it does not reflect inhibition or substrate activity. It is not considered when classifying the chemicals.
- "Not Determined" outcomes for compound activity are often due to the cytotoxic effect of chemicals. These entries are not taken into consideration when classifying.
- Records where the "relation" is annotated with the '>' or '<' signs, instead of '=', are generally not considered. However, if a continuous endpoint has a recorded activity over

one of the guideline thresholds (e.g., IC_{50} > 100 uM, % inhibition < 25%), the record will be assigned the value 0.

Case examples – classification for BCRP activity:

	ENDPOINT	RELATION	VALUE	INHIBITION CLASS	REASON	FINAL INHIBITION CLASS
**NOVOBIOCIN**	IC_{50}	=	66069.34 nM	1	authors	**1**
	IC_{50}	=	104000 nM	0	guideline threshold	
	IC_{50}	=	85100 nM	0	guideline threshold	
	IC_{50}	=	25000 nM	1	authors	
	K_i	=	100 nM	1	guideline threshold	
	Fold activity	=	3	1	guideline threshold	

**Majority vote is taken for the compound's class.*

	ENDPOINT	RELATION	VALUE	SUBSTRATE CLASS	REASON	FINAL SUBSTRATE CLASS
**METHOTREXATE**	Activity	=		1	authors	**1**
	K_m	=	1300000 nM	0	guideline threshold	
	Activity	=		1	authors (cited from literature)	
	K_m	=	680000 nM	0.5	authors	
	K_m	=	5700000 nM	1	authors	
	Activity	=		1	authors	

**Majority vote is taken for the compound's class*

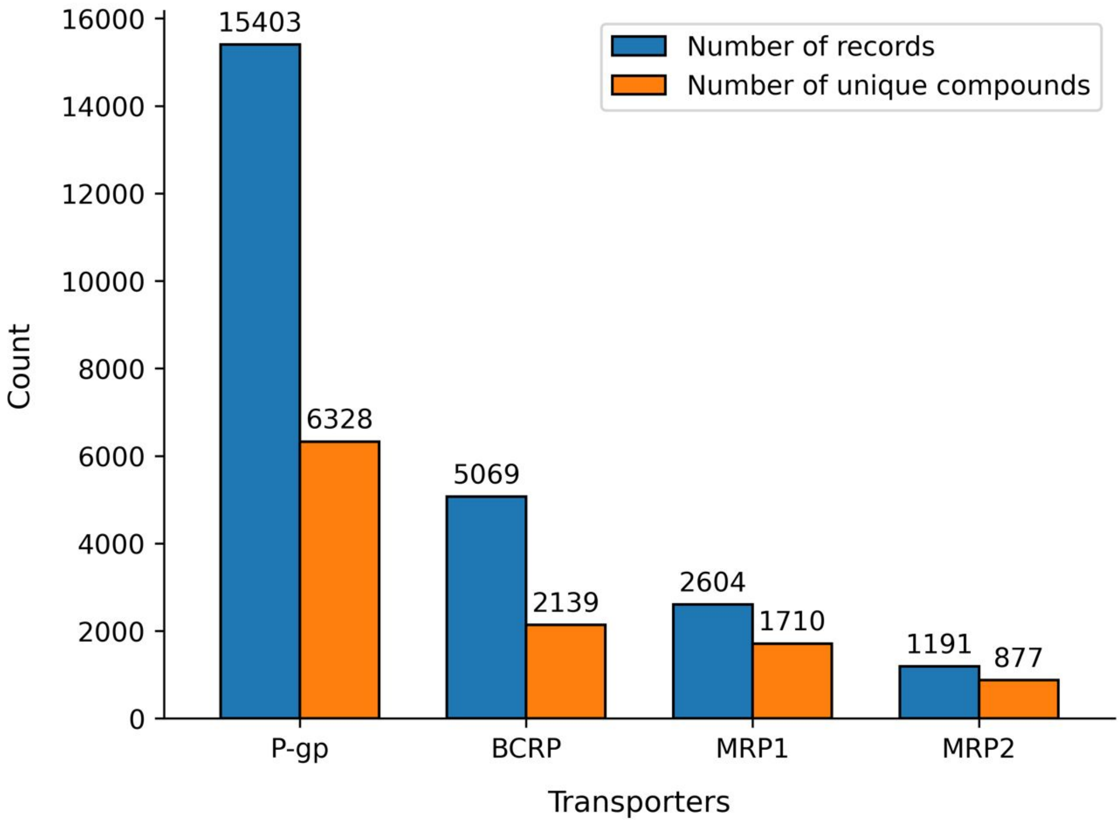

Figure S1. Bar plots illustrating the number of bioactivity records and unique compounds retrieved for P-gp, BCRP, MRP1, and MRP2.

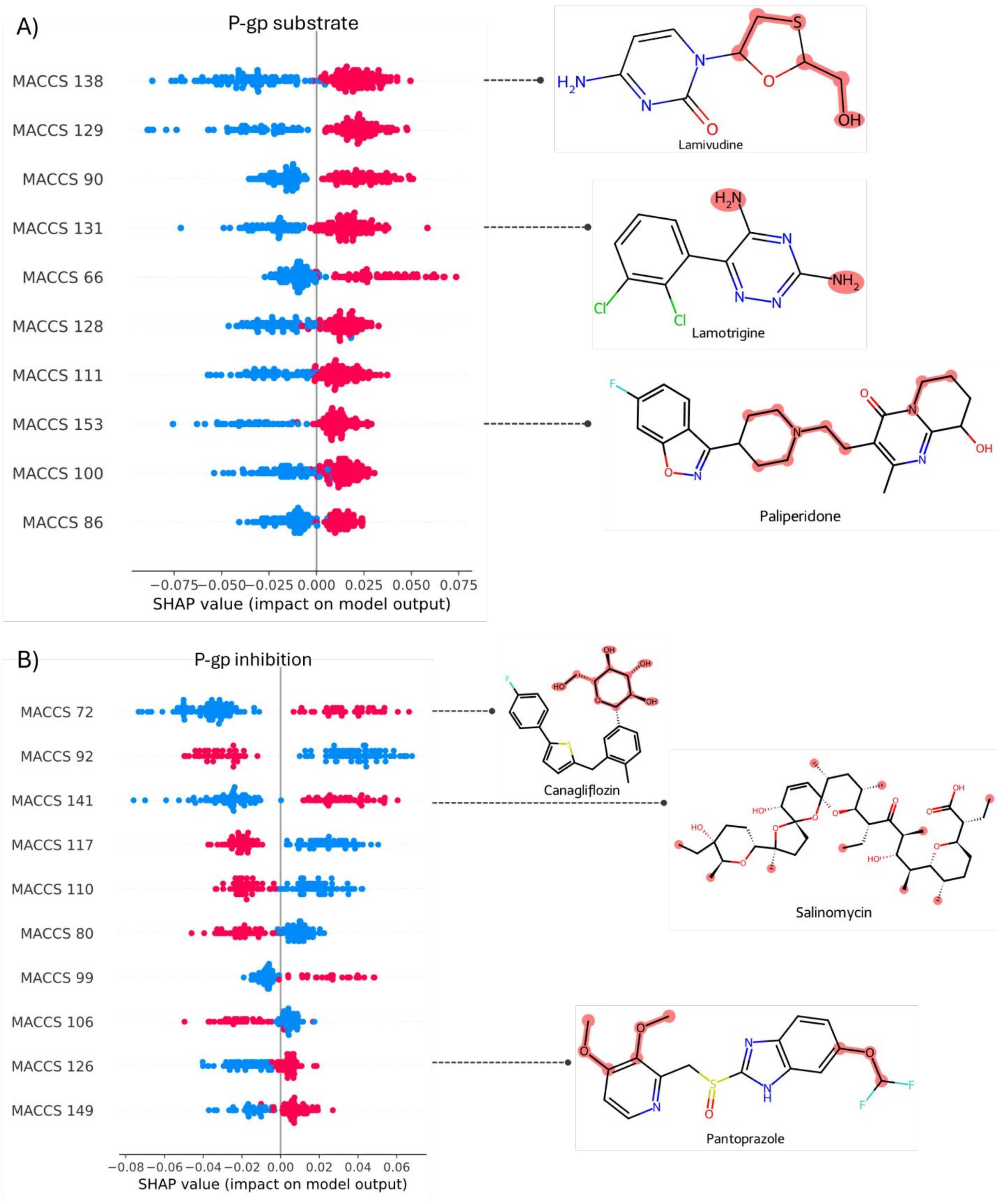

Figure S2. Top 10 MACCS descriptors ranked by SHAP values for the P-gp random forest A) substrate and B) inhibition models. Representative compounds with highlighted substructures illustrate the corresponding MACCS descriptors. The pink color in the beeswarm plot represents the presence of a descriptor (1) and the blue color represents the absence of the descriptor (0).

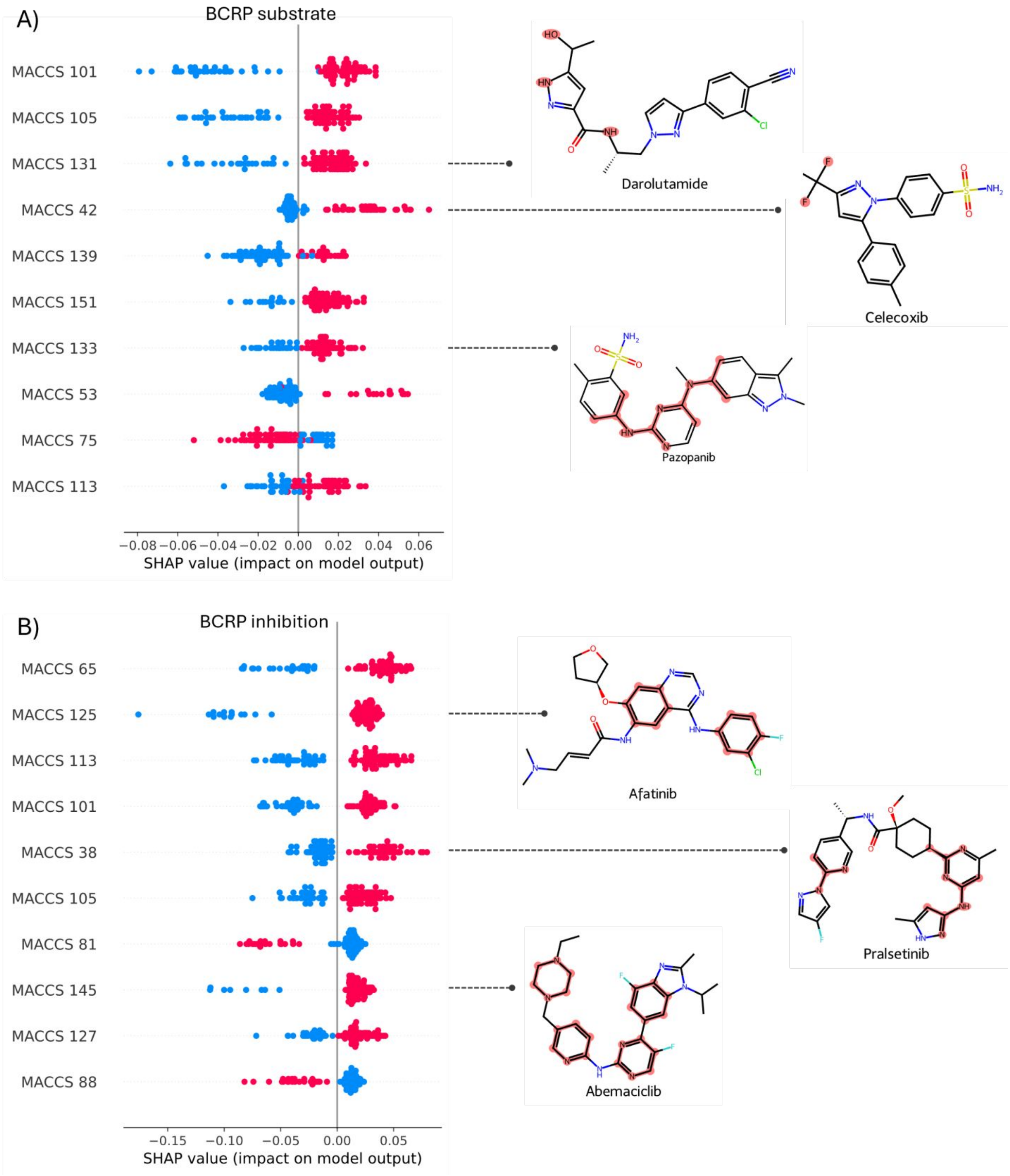

Figure S3. Top 10 MACCS descriptors ranked by SHAP values for the BCRP random forest A) substrate and B) inhibition models. Representative compounds with highlighted substructures illustrate the corresponding MACCS descriptors. The pink color in the beeswarm plot represents the presence of a descriptor (1) and the blue color represents the absence of the descriptor (0).

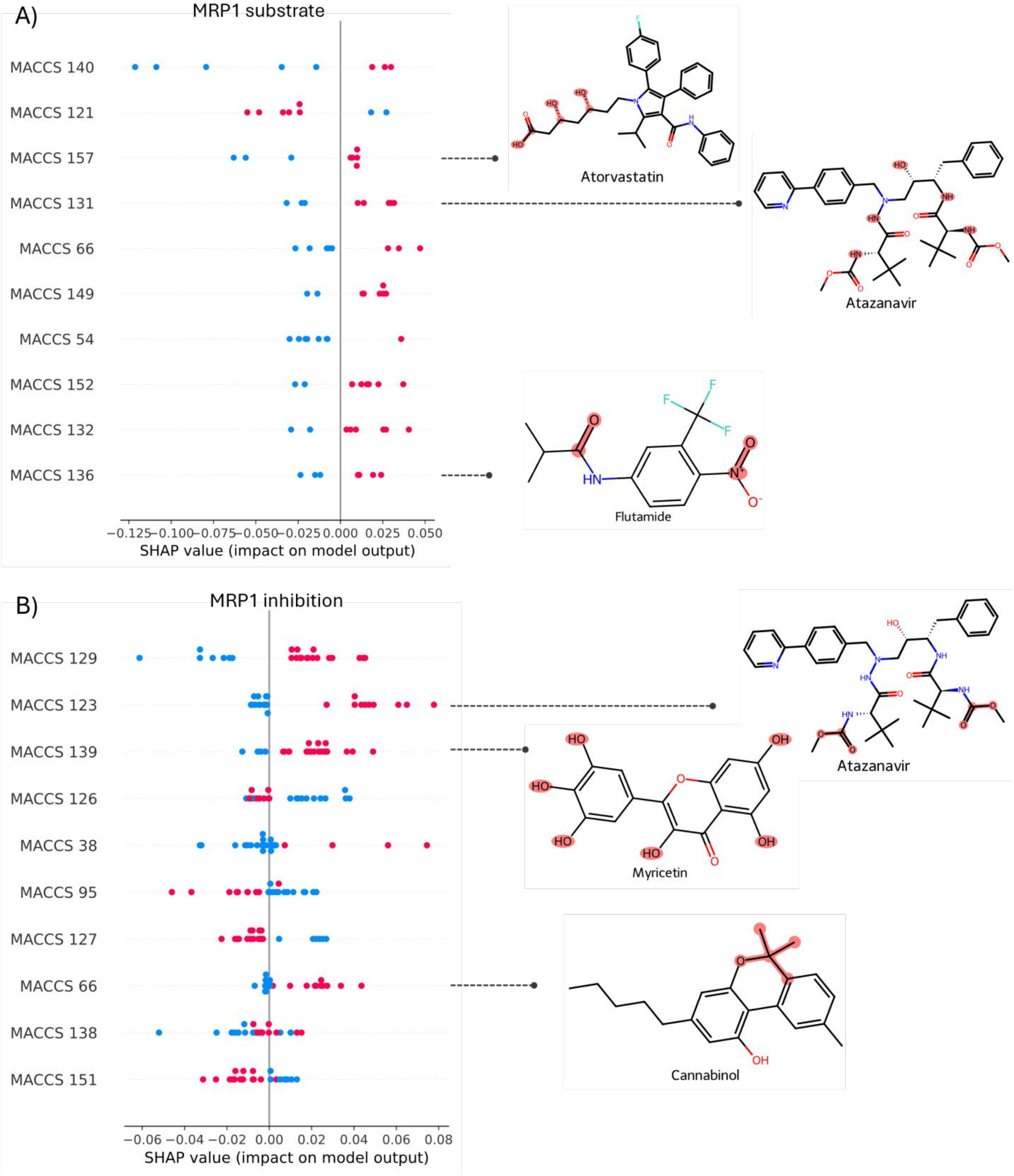

Figure S4. Top 10 MACCS descriptors ranked by SHAP values for the MRP1 random forest A) substrate and B) inhibition models. Representative compounds with highlighted substructures illustrate the corresponding MACCS descriptors. The pink color in the beeswarm plot represents the presence of a descriptor (1) and the blue color represents the absence of the descriptor (0).

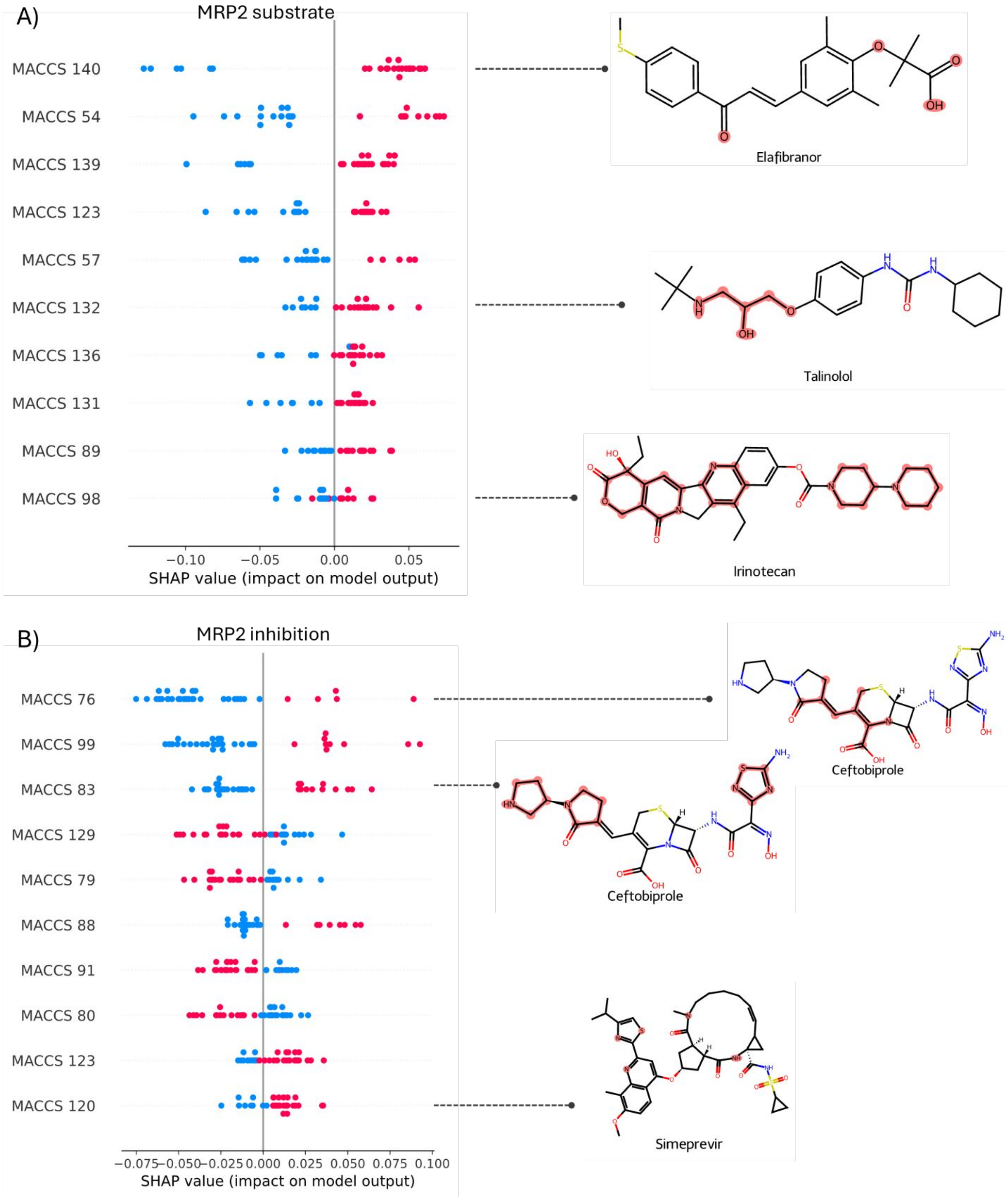

Figure S5. Top 10 MACCS descriptors ranked by SHAP values for the MRP2 random forest A) substrate and B) inhibition models. Representative compounds with highlighted substructures illustrate the corresponding MACCS descriptors. The pink color in the beeswarm plot represents the presence of a descriptor (1) and the blue color represents the absence of the descriptor (0).
